# Researches Anatomical and Physiological

**Published:** 1840-06

**Authors:** 


### REVIEWS.

---

Art. VI.—*Researches Anatomical and Physiological.* By John Davy, M. D. F. R. S. Assistant Inspector of Army Hospitals. In two volumes. London; Smith, Elder & Co., 65 Cornhill, 1839. pp. 935 8vo.

From the distinguished connexions, as well as the high standing of the author of this work (being as he is a brother of the late Sir Humphrey Davy,) and from the great length of time he has been engaged in the collection of materials for it, and in its composition, (some of it having been written nearly thirty years ago)—from these and other considerations, especially the wide and varied range of observation and research which it embraces, it ought to be hailed as a production of ample promise. And such we believe is the case in relation to it. Nor do we say that it will altogether fail to satisfy public expectation. Yet, that we ourselves are not a little disappointed in our hopes and anticipations respecting it, is not to be either denied or disguised. That, as a whole, it is much more voluminous and labored, than well conceived, judicious, and useful, we feel ourselves authorized, and deem it our duty as reviewers to assert. Yet are many sections of it characterised by a full sufficiency of interest and merit, to invite and reward an attentive perusal of them. The *whole host* of experiments it contains, though perhaps unnecessarily, not to say *exceptionably* numerous, we are not prepared to pronounce either inaccurate or unprofitable. Far otherwise. But the usefulness of *many* of them whether in a scientific or a practical point of view, (were it correct in us to make a distinction between *those qualities*,) we are unable to discover.

The work, as it now appears, does not possess the freshness of originality, the greater portion of it having been previously published at the periods when it was written, either as articles in the Transactions of the Royal Society, or in some of the minor Journals and Periodicals of the time. And, for making up those articles into volumes, the author assigns the following reasons:

> "I have recourse to re-publication, partly for the purpose of bringing together papers which are scattered in the transactions of Societies, and in Journals not easy of access; and partly with a view to alterations, corrections, and additions, which either after-reading, or further research has enabled me to make; and lastly, with the hope that the collection thus formed may prove of use, both to the medical student, and the medical inquirer."

The work consists of nearly fifty different papers, each of them treating of a subject more or less distinct from all the others. It need hardly be added, therefore, that to give the slightest notice of an entire collection, so extensive and multifarious, in a single review of the customary length, is altogether impossible. Hence we shall select, as the theme of our analysis and remarks, a single article entitled,

"*An account of some Experiments and Observations on the Torpedo.*"

And even of this our consideration must be limited; and to render it as free as possible from mistake and misrepresentation, we shall allow our author, in most instances, to speak for himself. In the following clause he states succinctly the defective condition of the knowledge possessed by naturalists and physiologists, respecting the phenomena exhibited by the torpedo, previously to the commencement of his own investigation of the subject, and makes known the points designed to be elucidated, by the experiments he instituted, in the course of his researches.

"I may premise that when I entered on the enquiry, it had not been ascertained, that the electricity of the torpedo, considering its peculiar influence to be electrical, had either the power of acting chemico-electrically, in separating the elements of compound bodies; or magnetically, either in affecting the needle in the multiplier, or in imparting magnetism to iron; or lastly of generating or producing heat; points to which my experiments were particularly directed, and with positive and successful results."

Before proceeding farther in our examination, we shall refer to a circumstance which, in our estimation, increases not a little the interest attached to the paper we are considering. It was written by its author at the request of his dying brother, Sir Humphrey Davy, president at the time of the Royal Society. That request was conveyed in the following letter, which beautifully and affectingly discloses the expiring philosopher's "ruling passion strong in death."

*Rome, Feb. 25th,* 1829.

"My dear John,

"If I had not had this attack, it was my intention to have gone to Fumicina, or Civita Vecchia, to make some experiments on the torpedo. I hope you will take up this subject, which, both as a comparative anatomist and chemist, you are very capable to elucidate. You will see my paper on the torpedo, in the manuscript book which I have left in Mr. Tobin's hands.* It was my wish to have exposed an unmagnetised needle to the continued shocks of a torpedo in a metallic spiral, making the metallic communication perfect with both electrical organs. There is in my little box, an apparatus which I hope you will use. Large living torpedos may be procured at Fumicina, or Civita Vecchia. The shock from a very small jar will make a needle magnetic, provided it is entirely passed through the metallic conductors; but I did not find this effect when there was any interruption by water. There are many things worth attending to in the two kinds of torpedinal fishes found here—the tremula and occhiatella. Pray do not neglect this subject, which I leave to you as another legacy. God bless you, my dear brother, your affectionate Friend,

"H. Davy."

The commencement of the writer's investigation of this curious and well selected subject, we shall communicate to our readers in his own words.

"*Experiments on the Electricity of the Torpedo.*

"From the preceding letter, it appears how desirous my brother was of trying the effect of the shock of the torpedo on a needle placed in a spiral wire. The result, he was of opinion, would be conclusive as to the nature of its electricity,—that is, whether it should be considered distinct, and of a peculiar kind, or merely a variety of common electricity, or at least analogous to kinds already known.

"Anxious to make this trial, I had an apparatus in readiness, which, with common electricity, I had found to answer extremely well. It consisted of a fine copper spiral wire, (the spring of a bracer) about one inch and a half long, and one-tenth of an inch in diameter, containing about one hundred and eighty convolutions, and weighing about four grains and a half. This was inserted into a glass tube, just large enough to receive it, and secured by corks.† The wire passed through the cork at each end, and was connected with

* This, his last paper and communication to the Royal Society, was published in the Philosophical Transactions, for 1829; it was written in the preceding October; its results were entirely negative.

† The apparatus referred to by my brother, in his letter, did not answer so well: the spiral was of silver wire wound round a glass tube.

strong wires with glass handles for the purpose of contact. The wire intended to be applied to the under surface of the fish was one-twenty-fifth of an inch in diameter; that intended for the upper surface was stiffer, being one-fourteenth of an inch in diameter, and its greater strength was useful, as it was necessary to employ it occasionally with some force to rouse the fish when averse to give a shock. The first trial I had an opportunity of making with this apparatus, was successful. It was on the 3rd of Sept. 1831, at eleven o'clock at night.* The fish used was a small one, about six inches long; it had been just caught in a hand-net, and immediately put into salt water, and was very active. A needle perfectly free from magnetism, was introduced into the spiral, and there confined by the corks, and the spiral was carefully connected with the insulated wires for contact. The fish for the experiment was placed in a glass basin, and was barely covered with water. One wire was applied to the under surface of the electrical organ, and the other to its upper surface, and contacts were made at intervals during about five minutes, when the fish seemed much exhausted by its exertions. On taking the needle out, and bringing it near some fine iron filings, it was found to be magnetic, and powerfully attracted them. This experiment I have repeated many times with fishes of different sizes, some larger and smaller, and with the same result whenever the fish has been active, and the contacts similarly made.

"The next trial instituted on the electricity of the torpedo was on the multiplier; it was one which belonged to my brother, the needle was poised on a pivot, and was not very sensible. The precaution was taken to insulate the instrument well, by smearing with sealing wax the feet of the stand supporting the coil. The same wires for contact were used in this as in the former experiments, and the junctions were carefully made. Applying one wire to the under surface, and the other to the upper surface, using the fish first mentioned, and after an interval of only two hours, I succeeded in obtaining decisive results; the first shock had a powerful effect, the needle made half a revolution: and other trials were in

* It may appear singular that this experiment was not made before. The explanation is easily made.—On my return from Rome to Malta, in June, 1829, I was assured that the torpedo is not known in the latter place. It was not until the summer of 1831 that I found out that I had been misinformed, and that, with a little trouble, the fish may be procured alive at all seasons of the year.

accordance. The needle, by active fishes, was generally thrown into violent motion, occasionally describing nearly a circle, and even by the feeblest it was distinctly affected. I have met with no instance of a fish which had the power of magnetising a needle in the spiral wire, failing to move the needle in the multiplier; but I have met with more than one example of a fish, whose electricity was equal to the latter effect, and not to the former."

Again says our author:

"I thought it possible, that by insulating the torpedo on a plate of dry glass, and wiping its circumference dry, and smearing it with oil, that the galvanometer might be affected. But in this, too, I have been disappointed; not even in flame, when the interruption of the circle has been only just visible, has any effect on the instrument been produced.*

"Mr. Farady, in the Third Series of his Experimental Researches on Electricity, states that he has little or no doubt, were Harris's electrometer applied to the torpedo, the evolution of heat would be observed.† I have made very many experiments on this subject, completely establishing Mr. Farady's anticipation. The instrument employed was similar to that described by Mr. Harris in the Philosophical Transactions for 1827, differing merely in the wire passed through the small globe being exceedingly fine, and of platina, formed after Dr. Wollaston's method;‡ in having a small stop-cock for regulating the height of the spirit in the stem; and in using as small a quantity of spirit as possible. The small

---

* Since the above remarks were first made, M. Matteucci has obtained sparks from the torpedo, and Mr. Farady from the Gymnotus,—not indeed directly, as Mr. Walsh was supposed to have done, but by means of a "magneto-electric coil," the object of which, it has been hypothetically said, is to prevent the electrical principles from neutralizing themselves directly through the conducting matter adjoining; and to force them to re-unite at a distance, by traversing the thin stratum of air in which the spark was taken. Bibliothèque Universelle de Genève, June, 1836, p. 387. The preceding part of this note was written in December;—I have since learned (now January 26, 1839,) that a spark has been taken direct from the Gymnotus, in the Adelaide Gallery, and that it was accidentally observed in making an experiment, instituted with a different object in view. My authority for this singular occurrence, and one so difficult of explanation, is the very intelligent superintendent, Mr. Bradley.

† Philosophical Transactions, 1813, p. 46.

‡ Philosophical Transactions, 1813, p. 114.

globe or bulb of the thermometer was defended from the variable temperature of the surrounding air, by being included in a wooden box. The delicacy of this instrument was so great, that the spirit in the stem was not only moved by a single spark of the electrical machine, but even very distinctly by the electricity of a single voltaic combination, composed of a copper and zinc wire, the former 1-25th of an inch in diameter, the latter 1-50th, excited by dilute sulphuric acid.

"This instrument was strongly affected by active fish, and even distinctly by weak ones;—indeed, occasionally, when it formed part of a circle in connexion with a galvanometer, I have seen it affected alone, the galvanometer affording no indication of the passage of the electricity. Using two air-thermometers of the same construction, each connected with the wires for contact at one end, and with the galvanometer at the other, the heating effect of the electricity of the torpedo has been apparently diminished, and even more distinctly diminished on adding to the circle another link of very fine platina wire. And at the same time its influence on the galvanometer has been diminished, and its power of imparting permanent magnetism to a needle placed in a spiral, both forming part of the circle. When heat has been applied to the entire link of platina by means of a spirit lamp, so as to render it red hot, the diminution of effect disappeared; and equally so, as well as I could judge from many experiments, whether acting on the thermometer, the galvanometer, or the needle in the spiral."

But we can no farther report Dr. Davy's experiments. Our space does not permit us to go into detail. To become acquainted with those experiments (and they are well worth the trouble of forming an acquaintance with them,) the reader must resort to the article before us, and be content for the present with a narrative of results. Our author continues:

"The tests or indications of electricity of the torpedo, at present known, are six in number; namely, the physiological effect, as the sensation it imparts is sometimes called; the chemical effects, as the precipitation of iodine, the decomposition of water, &c.; its effects on the thermometer, on the galvanometer, and on steel in the spiral. These different tests, in point of delicacy, I am inclined to believe, are in the order in which they are enumerated. That the two first should be placed highest, and that sensation should have the precedence, the experiments which I have made appear to prove independently of all analogy."

After giving a long and circumstantial anatomical description of the "*electrical organs*" of the torpedo, into which the nervous tissue enters, as the leading element, and subjoining a detailed account of the experiments performed by him, the Doctor communicates, in the form of results and inferences, what he frankly denominates "*theoretical remarks.*" Some of these are as follows, and deserve, in our opinion, the serious consideration of all philosophical naturalists and physologists.

"The experiments which I have detailed on the electricity of the torpedo, confirm those of Mr. Walsh, made in 1772, shewing its resemblance to common electricity. They moreover show, that like common electricity, and voltaic electricity, it has the power of giving magnetic polarity to iron, and of producing chemical changes. In these its general effects, it does not seem to be essentially peculiar, but as much allied to voltaic electricity, as voltaic electricity is to atmospheric, or atmospheric electricity is to that produced by contact or friction. When we examine more minutely its phenomena or effects, in relation to these different kinds or varieties of electricity, certain points of difference occur.

"Compared with voltaic electricity, its effect on the multiplier is feeble; its power of decomposing water and metallic solutions is inconsiderable; but its power of giving a shock is great, and so also is its power of magnetising iron.

"Compared with common electricity, it has a power of affecting the multiplier, which under ordinary circumstances, common electricity does not exhibit; its chemical effects are more distinct; its power of magnetising iron,* and giving a

* There is this difference when two spirals are used, one connected with the inside of a Leyden jar, and the other with the outside; a needle in each, similarly placed, acquires opposite polarities, the north pole in one being where the south pole is in the other; whilst in the instance of the torpedo they accord, so that a line of needles, passing from one side of the electrical organ to the other, would exhibit a succession of similar poles shock appear very similar; its power of passing through air is infinitely less; as is also its power of producing heat.

"There are other points of difference; I allude chiefly to the results obtained in the experiments already described, in which the metalic communication was interrupted by a strong solution of salt. In this instance the full power of the fish appeared to pass; water was decomposed, a shock was received, needles were magnetised, and the multiplier was affected. When the same experiment was made on the electrcity excited by the small voltaic combination of a single plate of copper and zinc, each less than an inch in length, and half an inch in breadth, immersed in an acid, neither water was decomposed, nor was the multiplier affected. When it was made on the electricity of the electrical machine, by means of a Leyden jar, all the effects were witnessed, excepting the motion of the multiplier, and the order of succession of poles in the needles magnetised in the spirals. How are these differences to be explained? Do they admit of explanation similar to that advanced by Mr. Cavendish, in his theory of the torpedo, and which has recently been ably advocated by Mr. Farady? or are they more in accordance with the idea which my brother was disposed to adopt, that the electricity of the torpedo and of the other electrical fishes is peculiar,—a power *sui generis?* or, lastly, may we suppose, according to the analogy of the solar ray, that it is not a single power, but a combination of powers?

"The first opinion, which is commonly received, is supported by the majority of facts detailed in the preceding pages. The circumstance principally hostile to it, at least in appearance, is the interruption of the torpedinal electricity by the smallest quantity of air, and its want of the power of attraction and repulsion in the air.

"These peculiarities are seemingly in favour of the second opinion, that the electricity of the torpedo is specific and peculiar. But till the opposite surfaces of the electrical organs can be perfectly insulated, so that no easier mode of communication is afforded than through air, they can hardly be considered of much weight.*

"The third opinion may be indulged in as an hypothesis; as a guide to research it may not be useless. It applies, how-

* In the experiments in which I attempted to insulate the surfaces, by means of oil, the probability is, that I failed, and that a communication continued, if not by the outer surface of the skin, at least by its inner; indeed, the attempts to insulate these organs, in the manner desired, seems to be almost hopeless.

ever, almost as much to other varieties of electricity as to that of the torpedo; all of which, it is possible, may be compounded, or owe their various effects to the union of several powers, or ethereal fluids, and their peculiarities compared one with another, to the predominance in various degrees of these fluids. What is known of the solar ray is not unfavourable to such an opinion; and the history of physical science, in relation to elementary ponderable matter, may give rather encouragement to the notion.

"As regards the mode of production, or the cause of the electricity of the torpedo, it is unavoidably enveloped in great mystery. Like animal heat, and the light emitted by certain animals, and I may add, the secretions of animals generally, it appears to be the result of living action; and connected with a peculiar and unusually complicated organization. All the attempts have been vain, which I have made, directed to obtain electrical excitement in the fish, after it had been deprived of life.

"The observations which I have detailed relating to its anatomical structure, show a complicated adaptation of parts—nerves of unusual magnitude ramifying between apparently insensible columns, saturated with a bad conducting fluid; muscles surrounding these columns, and fitted to compress them; and a system of mucous glands and tubes adjoining, well adapted to be the medium of electrical communication in the surfaces to which they belong.

"When we consider this structure, it is an easy matter to trace rude analogies between it and the pile of Volta, or between its columns, and a battery of Leyden jars, such a battery as was formed by Mr. Cavendish, for imitating the electricity of the torpedo, composed of a large number of jars of very thin glass feebly charged. But these analogies seem to help very little, if at all, towards the solution of the great difficulty: the question remains unanswered, what is the cause or source of the electricity? Here analogy fails entirely; none of the ordinary modes of excitement appear to be at all concerned—neither friction, nor chemical action, or change of temperature, or change of form. Let us consider for a moment a small torpedo in an active state. The smallest which I have employed in my experiments weighed only 410 grains, and contained only 48 grains of solid matter; its electrical organs weighed only 150 grains, and contained only 14 grains of solid matter,—for to this they were reduced by thorough drying. Yet this small mass of matter gave sharp shocks, converted needles into magnets, affected distinctly the multiplier, and acted as a chemical agent, effecting the decomposition of water, &c. *A priori*, how inconceivable that these effects could be so produced! This fish was about ten days in my possession, during the whole of which time it ate nothing, and its bulk was hardly sensibly altered; and every day it exercised its electrical powers, and to the last they appeared almost as energetic as when it was fresh from the sea. This adds, if possible, to the difficulty of explanation. That this mysterious function is intimately connected with the nerves, and in a manner more striking than all ordinary secretions, is manifest. Beyond this conclusion, all is darkness. We have not, as we have in the doctrine of animal heat, advanced another step—we have not been able to connect it with changes in the electrical organs, as analogous to known sources of electricity, as the changes which take place in the lungs in respiration are to the known sources of heat or combustion. The attainment of this step is a great desideratum, and beyond it, probably, we shall never be able to proceed.

"Without reverting to the conjectures, which in passing I have offered on the subserviency of the electricity of the torpedo in an auxiliary manner to digestion, respiration, and the secretion of mucus, I may remark that its chief use appears to be for purposes of defence—to guard it from its enemies, rather than to enable it, according to vulgar opinion, to destroy its prey, and provide itself with food. Small smelts, which were kept in the same vessel with torpedos, appeared to have no dread of them, and I believe they fed on their mucus. And, in an experiment in which, in a confined space, an active torpedo was excited to give shocks, a smelt which was with it was evidently alarmed, and once or twice exposed to the shock, leapt nearly out of the vessel; but was not injured by the electricity. In confirmation, I may add that the electric power of the young fish, which most requires it for its protection, is, as already observed, proportionally very much greater than that of the old, and can be excited without exhaustion and loss of life much more frequently. After a very few shocks, most of the old fish which I have had have become languid, and have died in a few hours; whilst young ones from three to six inches long have remained active during ten or fifteen days, and have never failed to shew the effects I have described."

Such are the amount and character of the analysis which we find it expedient give, at present, of our author's paper respecting the torpedo. That the subject is not only curious in itself, but highly interesting to all the votaries of physical science, especially to that portion of them who are earnest inquirers into the laws and habitudes of living matter, will not be denied. And should any one ask, whether it is also practically useful? we would promptly and positively reply in the affirmative—provided it be correctly and thoroughly understood. With whatever point in nature it may be connected, every truth, when fully known, is more or less practically useful, because it increases in some way the power and efficiency of those who possess it—conditioned that it be suitably and judiciously applied. This is as true, as that every dollar a man possesses makes a part of his wealth, and fits him, if properly employed, to act to better and more useful effect. This is but another version of the oft-quoted Baconian maxim that "knowledge is power."

The article before us is a paper on comparative anatomy and physiology. And the usefulness and importance of that study are now universally felt and acknowledged, by every physician who is worthy of his profession. So true is this, that, without some acquaintance with the anatomy and physiology of the inferior animals, the knowledge of human anatomy and physiology is necessarily defective. Were this position doubtful, it might be illustrated and confirmed by instances innumerable.

In the organization of living matter, the nervous tissue, including the brain and spinal cord, are at once the leading and commanding element. Were other considerations therefore wanting, the mere fact, that our author's paper tends to shed light on some of the functions and uses of that tissue, testifies to its importance. In examining that paper then, we have been contributing to the diffusion of *practical* knowledge. In future numbers of the Journal, we shall probably give analyses of some of the Doctor's other papers, especially of one on the "blood," and of another on "animal heat"—both of which we think productions of interest and merit. C. C.

